# Correlation between ITGB2 expression and clinical characterization of glioma and the prognostic significance of its methylation in low-grade glioma(LGG)

**DOI:** 10.3389/fendo.2022.1106120

**Published:** 2023-01-13

**Authors:** He Liu, Jiao Wang, Tao Luo, Zhiming Zhen, Li Liu, Yalan Zheng, Chaobin Zhang, Xiaofei Hu

**Affiliations:** ^1^ Department of Radiology, Southwest Hospital, Third Military Medical University (Army Medical University), Chongqing, China; ^2^ Institute of Pathology and Southwest Cancer Center, Southwest Hospital, Third Military Medical University (Army Medical University) and Key Laboratory of Tumor Immunopathology, Ministry of Education of China, Chongqing, China; ^3^ Department of Digital Medicine, School of Biomedical Engineering and Medical Imaging, Third Military Medical University (Army Medical University), Chongqing, China; ^4^ Southwest Eye Hospital, Third Military Medical University (Army Medical University), Chongqing, China; ^5^ Department of Nuclear Medicine, Southwest Hospital, Third Military Medical University (Army Medical University), Chongqing, China

**Keywords:** ITGB2, low-grade glioma, immune cell, methylation, survival

## Abstract

**Introduction:**

Glioma is the most common primary tumor in the brain.Integrin beta 2(ITGB2) is a member of the leukocyte integrin family (leukocyte integrin), participating in lymphocyte recycling and homing, cell adhesion, and cell surface-mediated signal transduction. However, few studies on ITGB2 in gliomas have been reported yet.This study first discussed the relationship between ITGB2 expression and clinical characterization of glioma and the prognostic significance of its methylation in low-grade glioma.

**Methods:**

We collected Clinical data and transcription of glioma patients from TCGA, CGGA, and Rembrant datasets to analyze the differential expression of ITGB2 mRNA in glioma tissues and normal tissues. The box polts to evaluated the expression patterns of ITGB2 in different molecular subtypes. Receiver operating characteristic curve (ROC) were used to evaluate and verify the reliability of the model. Kaplan-Meier survival curves to evaluated the relationship between the level of ITGB2 mRNA expression and overall survival (OS). Using cox regression analysis to verify the ability of ITGB2 as an independent predictor of OS in glioma patients. We use TIMER to analyze and visualize the association between immune infiltration levels and a range of variables. The methylation of GBMLGG patients were obtained from the TCGA database through the biological portal.

**Results:**

ITGB2 can be a potential marker for mesenchymal molecular subtype gliomas. COX regression analysis shows that ITGB2 is an independent predictive marker of OS in malignant glioma patients. Biological processes show that ITGB2 has involved glioma immune-related activities, especially closely related to B cells, CD4+Tcells, macrophages, neutrophils, and dendritic cells. ITGB2 is negatively regulated by ITGB2 methylation, resulting in low expression in LGG tissues. Low expression of ITGB2 and high methylation indicate good OS in patients with LGG. The ITGB2 methylation risk score (ITMRS) obtained from the ITGB2 methylation CpG site can better predict the OS of LGG patients. We used univariate and multivariate cox regression analysis of methylationsites, used the R language predict function to obtain the risk score of these ITGB2 methylation sites(ITMRS).

**Discussion:**

ITGB2 can be used as a potential marker of mesenchymal molecular subtypes of gliomas and as an independent predictive marker of OS in patients with malignant gliomas. The ITMRS we established can be used as an independent prognostic factor for LGG and provide a new idea for the diagnosis and treatment of LGG.

## Introduction

Although glioma accounts for only 2% of adult cancer and 46% of all intracranial tumors, it is still the most common primary tumor in the brain (1). The World Health Organization (2016, Revised edition) classifies diffuse gliomas as WHO II-IV (2). There are significant differences in the Pathological morphology of the tumor(such as collagen fiber content and morphological diversity), tumor development, and patient prognosis. Among them, glioblastoma (GBM, WHO IV) is the most aggressive glioma with the worst prognosis (3), and the 5-year survival rate is only 9% (4). The five-year survival rate of grade WHO III gliomas are only 30%. Clinically, Grade WHO III and IV are collectively referred to as malignant gliomas because of their strong invasiveness and short survival time (5). Although low-grade gliomas (LGG, WHOII) have comprehensive treatments such as neurosurgical resection, chemotherapy, and radiotherapy, the 5-year survival rate is 50% (6). Due to its inevitable drug resistance, tumor recurrence, and the risk of rapid development of GBM (7). There is an urgent need to find a new and highly accurate biomarker to provide new ideas for the treatment and prognosis prediction of patients with GBM or LGG.

Integrin beta 2(ITGB2), also known as CD18/LFA-1, is a member of the leukocyte integrin family (leukocyte integrin). It binds to various α chains to form different integrin heterodimers. Integrin is an essential protein on the cell surface, participating in lymphocyte recycling and homing (8), cell adhesion, and cell surface-mediated signal transduction (9, 10). ITGB2 plays a crucial role in the immune response, and the gene defect can lead to leukocyte adhesion deficiency. It has been reported that the lack of ITGB2 plays a positive role in preventing autoimmune diabetes (11), and the high expression of ITGB2 promotes the migration and invasion of breast cancer (12). In nasopharyngeal carcinoma, the high expression of ITGB2 is related to the low overall survival rate and progression-free survival rate (13). ITGB2 is involved in binding lymphocytes to brain tumor tissue and subsequent migration (14). ITGB2 is vital in various diseases and cancers, especially tumor immunotherapy and migration. However, few studies on ITGB2 in gliomas have been reported yet, remaining clinical and prognostic significance unclear, especially in LGG.

This study first discussed the relationship between ITGB2 expression and different grades and types of gliomas. We verified the predictive function of ITGB2 to mesenchymal subtypes and the prognostic role of ITGB2 by using the Chinese Glioma Genome Atlas (CGGA), Rembrandt, and The Cancer Genome Atlas (TCGA) database analysis. Univariate and multivariate cox regression analysis explored the potential value of ITGB2 in the clinic. In addition, as the critical role of the immune microenvironment in the progression of LGG has attracted widespread attention (15–18), we have also mined the TIMER(Tumor Immune Estimation Resource) database of tumor immune estimation resources to evaluate the potential correlation between ITGB2 and LGG immune infiltration levels. The biological process ITGB2 involved was detected by gene enrichment analysis to study the mechanism of ITGB2 in LGG. Finally, through the analysis of TCGALGG methylation data, we obtained ITGB2 methylation risk score (ITMRS) using nine selected key ITGB2 methylation CpG sites. We evaluated the significance of ITMRS in the prognosis of LGG patients.

## Materials and methods

### Patients and data collection

First of all, we are on the Gene Expression Profiling Interactive Analysis (GEPIA) (19) website (http://gepia.Cancer-pku.cn/index.html) to analyze the differential expression of ITGB2mRNA in GBM and LGG tissues and normal tissues, and the relationship between the expression of ITGB mRNA and Overall Survival in patients with GBM and LGG. Then, we download the clinical and RNA-seq expression data from the mRNAseq_325 dataset in the CGGA database (http://www.cgga.org.cn/) for follow-up analysis. Download clinical and RNA-seq expression data from the Rembrandt database on the gliovis online website (http://gliovis.Bioinfo.cnio.es/). Clinical data, transcription, and methylation of GBMLGG patients were obtained from the TCGA database through the biological portal (20) (https://www.cbioportal.org/). The inclusion criteria were (1) patients with WHO grade II-IV and (2) patients with complete clinical and transcriptional data (CGGA:324, TCGA:607, Rembrandt: 139) were included in this analysis.

### Correlation analysis of immune infiltration

We use TIMER, a comprehensive website (https://cistrome.shiny-apps.io/timer/) (21), to automatically analyze and visualize the association between immune infiltration levels and a range of variables. The correlation of ITGB2 expression in six kinds of immune cells (CD4+T cells, CD8+T cells, B cells, neutrophils, dendritic cells, and macrophages) in GBM and LGG was analyzed. Using TISIDB (an integrated repository portal for tumor-immune system interactions), a database of tumor immunity database included 4176 records from 2530 publications and recorded 988 genes involved in anti-tumor immunity. The spearman correlation between ITGB2 and lymphocyte Immunostimulator and Immunoinhibitor related molecules in GBM and LGG was analyzed.

### Gene ontology enrichment analysis

The genes significantly related to ITGB2 expression were retrieved by using Pearson correlation analysis. The associated gene set was analyzed by using the GenecoDis website (22) (https://genecodis.genyo.es/). Besides, the hot map package of R Language is used to list genes related to ITGB2 expression positive height.

### Immunohistochemistry staining

Formalin-fixed samples were embedded in paraffin and sectioned at a thickness of 3 μm. The slides were deparaffinized and rehydrated, then incubated with a ITGB2 antibody (Sigma-Aldrich, HPA008877, 1:200). The mean density(integrated optical density(IOD) SUM/area) was calculated in five randomly selected fields using Image-Pro Plus 6.0 software.

### Statistical analysis

According to the median of ITGB2 mRNA expression in different data sets, the high expression and low expression groups of ITGB2 were established. Similarly, based on the median of ITGB2DNA methylation in TCGA-GBMLGG data sets, two ITGB2 hypomethylation and hypermethylation groups are established. Kaplan-Meier curve was used to evaluate the prognostic value of ITGB2 expression and ITGB2DNA methylation. The correlation between ITGB2 expression and ITGB2DNA methylation level was analyzed by Pearson correlation coefficient. In addition, we used univariate and multivariate Cox regression models to explore whether ITGB2 expression is an independent prognostic indicator for patients with LGG. The prediction performance of ITGB2 in the mesenchymal molecular subtype was evaluated by ROC curve analysis. Student t-test was conducted to explore the distribution of expression in different groups. Pearson correlation analysis was used to identify genes related to ITGB2 expression. R language packs (dplyr, stringr, survival, survminer, plyr, pheatmap, proc, and corcrac) are used for other statistical calculations and drawing data. All the differences were statistically significant at the level of P < 0.05.

## Results

### ITGB2 mRNA expression is upregulated in high-grade gliomas and downregulated in IDH1 mutation gliomas

**Figure 1:**
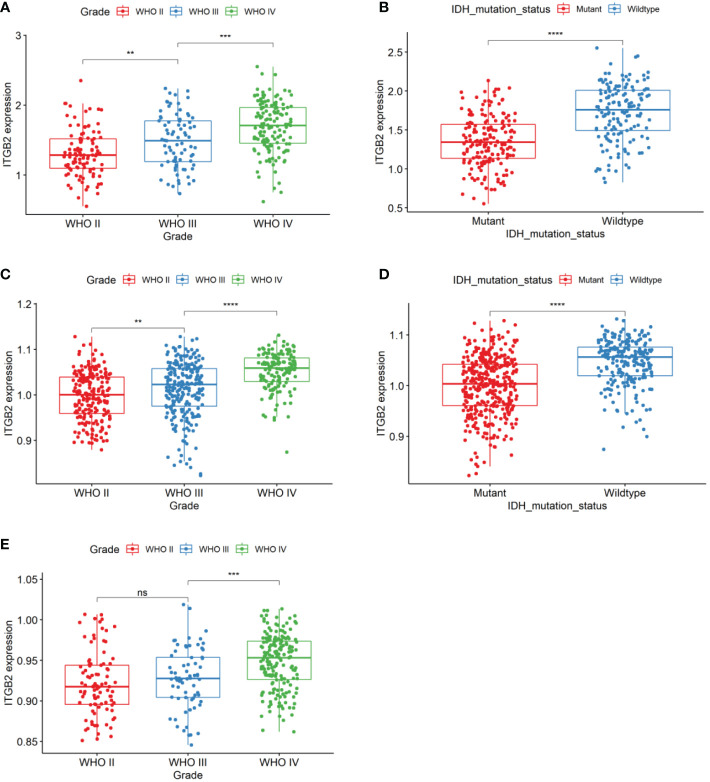
ITGB2 mRNA expression pattern in CGGA RNA-seq, TCGA-GBMLGG RNA-seq, and Rembrandt microarray datasets. **(A)** ITGB2 is enriched in high-grade gliomas in CGGA RNA-seq sets. **(B)** ITGB2 is enriched in IDH1 wt gliomas in CGGA RNA-seq sets. **(C, E)** ITGB2 is enriched in high-grade gliomas in TCGA-GBMLGG RNA-seq and Rembrandt microarray datasets. **(D)** ITGB2 is enriched in IDH1 wt gliomas in TCGA-GBMLGG RNA-seq and Rembrandt microarray datasets. ns, no significant differences; **p < 0.01, ***p < 0.001, and ****p < 0.0001.

**Figure 2:**
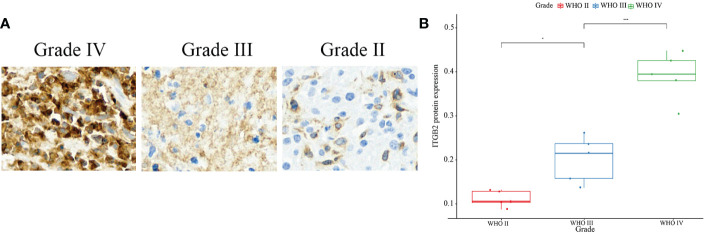
IHC staining of ITGB2. **(A)** IHC staining of ITGB2 in different grade, ×20. **(B)** Protein expression of ITGB2 in different grades of glioma(mean density,IOD SUM/area). *p < 0.05, ****p < 0.0001.

First of all, the clinical and RNA-seq expression profile data with DataSet ID as mRNAseq_325 were downloaded from the CGGA database. The phenotypic data were downloaded from the gliovis (http://gliovis.bioinfo.cnio.es/) database. **Figure 1A** showed that the expression of ITGB2 mRNA was positively correlated with tumor grade. In addition, the expression of ITGB2 in the isocitrate dehydrogenase 1 wild type (IDH1 Wt) group was higher than that in the IDH1 mutant (IDH1 Mut) group. **Figure 1B**). These findings were subsequently validated in TCGA GBMLGG’s RNA-seq dataset and Rembrant’s Microarray dataset (**Figures 1C–E**). The results showed that ITGB2 mRNA expression has a high correlation with tumor grade and IDH1 mutation in the CGGA database (p < 0.05). Subsequently, we randomly selected five patients with different grades of glioma to perform immunohistochemical experiments, and all patients voluntarily signed the informed consent. The results of immunohistochemistry (IHC) showed that the expression of ITGB2 protein (mean density, IOD SUM/area) was higher in high-grade gliomas (P<0.05), and there were statistically significant differences among different grades of gliomas (**Figures 2A, B**).

### ITGB2 is a potential marker for mesenchymal molecular subtype gliomas

**Figure 3:**
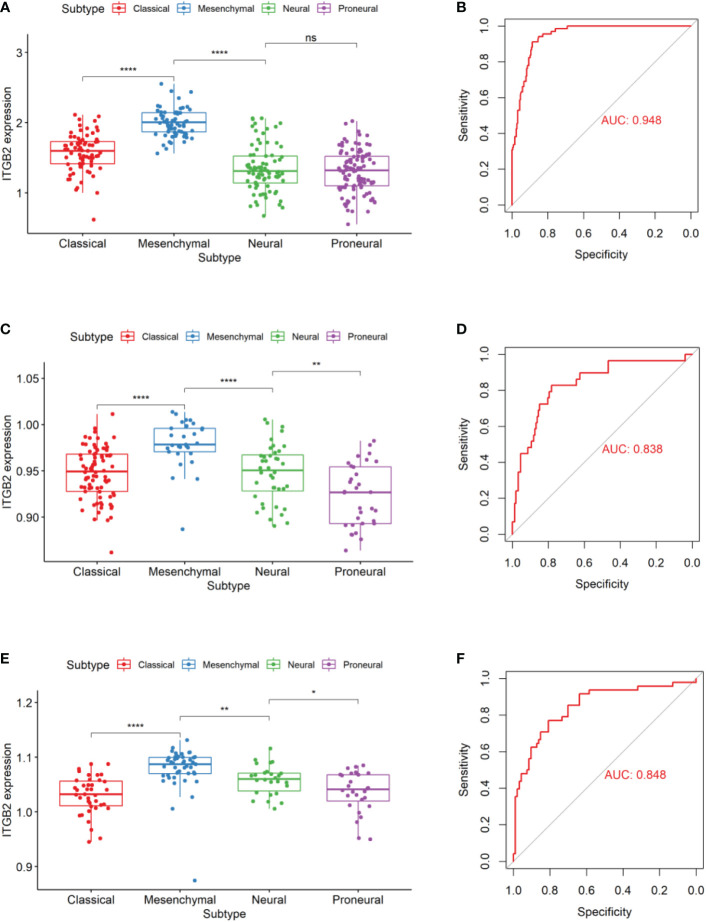
ITGB2 expression in different molecular subtypes of the TCGA transcriptional classification scheme in CGGA **(A)** and Rembrandt **(C)** and TCGA-GBMLGG **(E)** datasets.ROC curves of ITGB2 expression to predict mesenchymal subtype in CGGA **(B)** and Rembrandt **(D)** and TCGA-GBMLGG **(F)** datasets. ns, no significant differences; *p < 0.05, **p < 0.01, ****p < 0.0001.

In 2010, Verhaak’s team (23) divided glioblastoma into four subtypes using TCGA data sets. Through genomic mutation, copy number variation, and expression profile data, it was proved that epidermal growth factor receptor (EGFR), neurofibromin 1(NF1), and platelet-derived growth factor receptor alpha (PDGFRA)/isocitrate dehydrogenase 1 (IDH1) could be used as markers of classical, interstitial and proneuronal types, respectively. It provides a basis for targeted therapy of gliomas. So we evaluated the expression patterns of ITGB2 in different molecular subtypes. Results showed that the ITGB2 expression of mesenchymal subtypes was significantly higher than other subtypes in the RNA-seq set of CGGA and TCGA-GBMLGG and the Microarray data set Rembrandt (**Figures 3A, C, E**). We performed ITGB2 expression and receiver operating characteristic curve (ROC) analysis of mesenchymal subtypes in all grades of gliomas to verify this finding further. What is exciting is that in these three datasets, the area under the curves (AUC) are 0.948, 0.862, and 0.848, respectively (**Figures 3B, D, F**).

### ITGB2 is an independent predictive marker of OS in patients with gliomas

**Figure 4:**
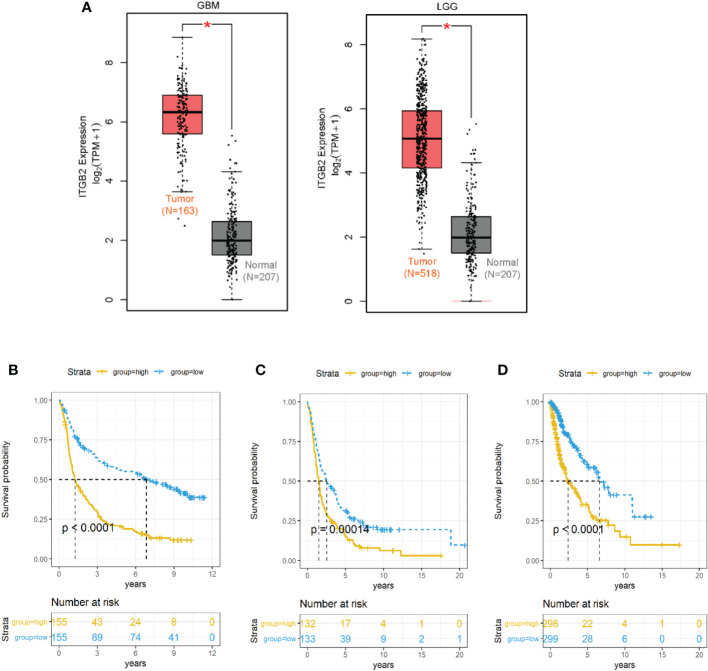
ITGB2 mRNA expression was related to clinical outcomes in gliomas. **(A)** ITGB2 mRNA is lowly expressed in LGG and GBM in TCGA dataset. **(B–D)** Kaplan-Meier estimates of survival for all grade patients in the CGGA RNA-seq **(A)** and TCGA-GBMLGG RNA-seq**(B)** and Rembrandt microarray set **(C)**. ITGB2 expression was negatively associated with OS of all grade gliomas (p< 0.05). *p < 0.05.

We used the GEPIA database to analyze the RNA sequencing data of 681 cases of TCGA tissues (518 LGG tissues and 163 GBM tissues) and 207 normal tissues of the GTEx plan. It was found that ITGB2 mRNA was highly expressed in both GBM and LGG tissues, while low expression was found in normal tissues (**Figure 4A**). Then, We used the median expression level of ITGB2 mRNA as the cutoff point to evaluate the relationship between the level of ITGB2 mRNA expression and survival time in different data sets. It was found that the patients with high expression of ITGB2 in CGGA RNA-seq had lower OS in all grades of gliomas (**Figure 4B** P < 0.0001). Similar results were also obtained in TCGA-GBMLGG RNA-seq set and Rembrandt Microarray data set (**Figures 4C, D**; for all grades of gliomas, p < 0.0001). Then, univariate and multivariate Cox regression analysis was performed in CGGA RNA-seq to verify whether the expression of ITGB2 was an independent prognostic factor (**Table 1**). Univariate regression analysis showed that ITGB2 (p < 0.001), grade (p < 0.0001), age (p < 0.0001), and IDH1 status (p < 0.0001) were each associated with OS. In multivariate regression, ITGB2(p < 0.0001)and grade(p < 0.0001)showed significant results. These results suggest that ITGB2 plays an important role in the occurrence and development of gliomas. Subsequently, biological function analysis should be carried out to verify our findings further.

**Table 1:** Univariate and multivariate Cox analysis in CGGA RNA-seq set.

Clinical factors	Univariate			P value	Multivariate			P value
	HR	95% CI			HR	95% CI		
		Lower	Upper			Lower	Upper	
Age	1.031	1.018	1.044	<0.0001	1.012	0.999	1.024	0.063
Gender	1.083	0.819	1.431	0.576				
IDH mutation	0.384	0.289	0.509	<0.0001	1.085	0.761	1.547	0.654
Grade	2.705	2.249	3.254	<0.0001	2.350	1.908	2.896	<0.0001
ITGB2	4.020	2.817	5.735	<0.0001	2.306	1.543	3.446	<0.0001

### ITGB2 is associated with immune functions in gliomas

**Figure 5:**
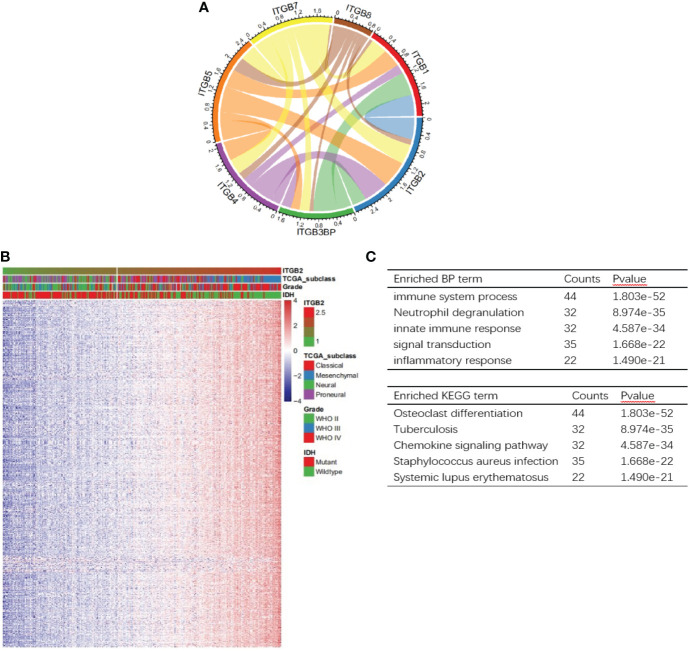
Analysis of biological processes related to ITGB2. **(A)** Correlation of ITGB2 with ITGB molecular in the CGGA RNA-seq set. **(B)** One hundred fourty genes positively related (R> 0.8) with ITGB2 expression. **(C)** The BP and KEGG analysis results show that ITGB2 expression is related to immune function of gliomas. Counts represents the number of genes enrichment.

In order to study the relationship between ITGB2 and other ITGB molecular families, we carried out Pearson correlation analysis on the CGGA RNA-seq set. It can be seen from **Figure 4A** that the expression of ITGB2 was significantly correlated with ITGB1, ITGB3BP, ITGB4, ITGB7, and ITGB8. To explore the biological process related to the expression of ITGB2 in gliomas, we conducted Pearson related analysis between ITGB2 expression and other genes in whole-genome gene profiling of 325 patients in the CGGA RNA-seq set. The results showed that 658 genes (R > 0. 6) were positively correlated with ITGB2 expression. Among them, 140 genes (R > 0. 8) were highly positively correlated with the expression of ITGB2 (**Figure 5A**). The Biological Process (BP) and KEGG analysis of the biological process of these 140 genes was carried out by using the GeneCodis website (https://genecodis.genyo.es). Results have been shown: The five most enriched biological process annotations were (1) Immune system process (p = 2.615e-52); (2) Neutrophil degranulation (p = 2.774e-36); (3) Innate immune response (p = 5.923e-34); (4) Inflammatory response (p = 7.096e-23); (5) Signal transduction (p = 1.743e-22), containing 44、33、32、23 and 35 genes from the query set, respectively. KEGG analysis includes Osteoclast differentiation, Staphylococcus aureus infection, Tuberculosis, Phagosome, and Neutrophil extracellular trap formation. In summary, all above results suggest that ITGB2 can affect glioma-related immune activity.

### Association of ITGB2 expression with tumor-infiltrating lymphocytes

**Figure 6:**
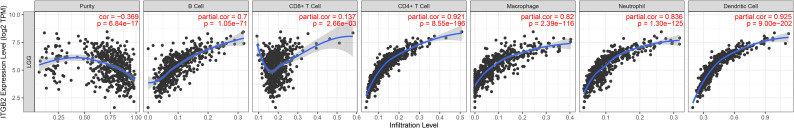
The expression of ITGB2 was related to a panel of gene markers of immune cells, including B cell, CD8+ T cell, CD4+ T cell, Macrophage, Neutrophil, Dendritic Cell.

The TIMER algorithm (https://cistrome.shinyapps.io/timer/) (21) was used to determine the relationship between the expression of ITGB2 and immune cell infiltration. According to the LGG and GBM cohort of TCGA, the expression of ITGB2 in LGG was negatively correlated with tumor purity (r = 0.369, P < 0.05). The expression of ITGB2 was positively correlated with B cells (r = 0.700, P < 0.05), CD4+T cells (r = 0.921, P < 0.05), macrophages (r = 0.820, P < 0.05), neutrophils (r = 0.836, P < 0.05) and dendritic cells (r = 0.925, P < 0.05). The expression of ITGB2 was weakly positively correlated with that of CD8+T cells (r = 0.137, P < 0.05). The expression of ITGB2 in GBM was negatively correlated with tumor purity (r = 0.559, P < 0.05) and CD8+T cells (r = 0.414, P < 0.05). The expression of ITGB2 was positively correlated with CD4+T cells (r = 0.435, P < 0.05), neutrophils (r = 0.390, P < 0.05) and dendritic cells (r = 0.562, P < 0.05). It was weakly positively correlated with B cells (r = 0.234, P < 0.05) and macrophages (r = 0.215, P < 0.05) (**Figure 6**). It is suggested that ITGB2 is closely related to immune cell infiltration in both LGG and GBM.

### The clinical and prognostic value of ITGB2 methylation CpG sites

**Figure 7:**
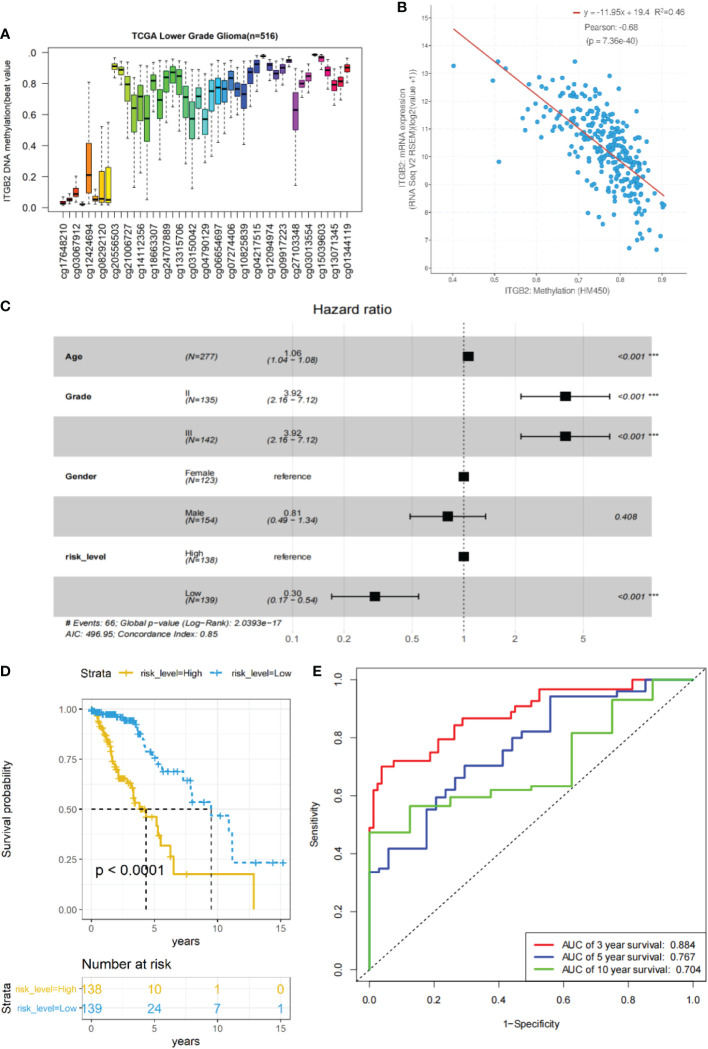
The relationship between ITGB2 expression and methylation in LGG tissues revealed by bioinformatic analysis. **(A)**The distribution of 45 ITGB2 DNA promoter CpG sites. **(B)** The expression of ITGB2 was negatively regulated by ITGB2 DNA methylation. **(C)** Forest plot of low ITMRS can be used as an independent risk factor in LGG patients from the TCGA-LGG dataset (P < 0.001). **(D)** survival analysis of LGG patients with a high ITMRS (ITMRS High) versus low ITMRS (ITMRS Low) in the TCGA LGG datasets. The P value was determined by the chi-square test between the two groups. **(E)** ROC curves of the prediction of 3-year survival、5-year survival、10-year survival with ITMRS in the TCGA LGG datasets.Risk level high and low represent ITMRS high and low. ***p < 0.001.

In order to further explore whether the methylation CpG site of ITGB2 also has clinical prognostic value. Using cBioPortal online database (http://www.cbioportal.org/), We found that there were significant differences in ITGB2 methylation in IDH WT and mutation gliomas (P < 0.0001, **Figure S1A**), The mRNA expression of ITGB2 was also significantly different in MGMT methylated and unmethylated gliomas(P < 0.0001, **Figure S1B**). Then, We evaluated whether ITGB2 methylation has clinical and prognostic value. We found 45 CpG sites of ITGB2 methylation, and there was a significant negative correlation between the expression of ITGB2 mRNA and ITGB2 methylation (r = 0.66, P < 0.0001, **Figures 7A, B**). Through univariate and multivariate cox regression analysis of 45 ITGB2 methylation sites, nine methylation sites may be independent risk prognostic factors were selected (**Figure S2**). Then we analyzed the prognostic value of these nine ITGB2 methylation CpG sites in patients with TCGA LGG. The Kaplan-Meier diagram shows that the high methylation level at the selected CpG site is associated with a more favorable OS in LGG patients. In order to better predict the prognosis of LGG patients, we used the R language predict function to obtain the risk score of these nine ITGB2 methylation sites(ITMRS). According to the median, we divided the ITMRS into two groups: high and low. **Figure 4C** shows that ITMRS low can be used as an independent risk prognostic factor. Then we analyzed the prognostic value of ITMRS in patients with TCGA LGG. Excitedly, ITMRS has a better prognosis in LGG patients than a single ITGB2 methylation CpG site predicts OS in LGG patients (**Figure 7D**). At the same time, we obtain the analysis of receiver operating characteristic curve(ROC) of the three-year, five-year, and ten-year survival predicted by ITMRS, and the area under the curve (AUC) was 0.884, 0.767 and 0.704, respectively(**Figure 7E**). The Kaplan-Meier diagram and ROC curves show that the low level of ITMRS is associated with a more favorable OS in LGG patients.

## Discussion

Glioma is a primary malignant tumor with high mortality rate (24). However, surgery and postoperative radiotherapy plus chemotherapy have improved the survival rate of glioma patients. However, the prognosis of most patients with gliomas is still poor (25). Therefore, there is an urgent need to find new and effective treatments that can increase the survival of glioma patients (26). Immunotherapy for gliomas is considered to have a bright future. Anti-glioma immunotherapy is a generic term that includes strategies designed to stimulate patients’ innate and acquired immune systems against gliomas and promote immune-mediated anti-glioma responses (27).

At present, the oncogenes related to the pathogenesis of gliomas have been identified and recorded (28, 29). There is few research on ITGB2 in gliomas. Only three articles related to gliomas have mentioned ITGB2 (30–32), but there is no in-depth study on the clinical and prognostic role of the ITGB2 gene. In our study, according to the molecular classification of glioma based on gene expression described by the TCGA network, the RNA-seq data set of CGGA, the RNA-seq set of TCGA-GBMLGG, and the Microarray data set of Rembrandt were divided into proneural type, neural type, classical type and mesenchymal type (23). It was found that the malignant degree of gliomas in the mesenchymal group was higher than that in other groups. ROC curve analysis showed that the expression of ITGB2 could highly predict mesenchymal subtypes, suggesting that ITGB2 could be used as a biomarker of mesenchymal subtypes. ITGB2 can significantly predict OS in all grades of gliomas(Since Rembrant’s Microarray dataset does not have IDH mutation status information, we cannot verify it in Rembrant’s Microarray dataset). Also, there were significant differences in mRNA expression levels among different grades and IDH1 status groups. Univariate and multivariate Cox regression analysis confirmed the crucial role of low expression of ITGB2 in the excellent prognosis of patients with LGG. In addition, we also verified the prognostic role of ITGB2 expression in two other data sets, and these results emphasized the promising prognostic value of ITGB2 expression in patients with LGG.

At the same time, our analysis showed that the expression of ITGB2 in LGG was significantly correlated with the level of immune infiltration. It was significantly positively correlated with B cells, CD4+T cells, macrophages, neutrophils, and dendritic cells. As far as we know, the ITGB2 gene encodes the integrin β chain, which binds to various α chains to form different integrin heterodimers. Integrin is an indispensable protein on the cell surface, which participates in cell adhesion and cell surface-mediated signal transduction. It encodes proteins that play an essential role in the immune response, which is also consistent with our analysis that ITGB2 is involved in biological processes such as the immune system process, neutrophil degranulation, and innate immune response, inflammatory response, signal transduction, and so on. These pieces of evidence suggest that ITGB2 participates in the regulation of tumor immune microenvironment mainly by regulating B cells, CD4+T cells, macrophages, neutrophils, and dendritic cells in LGG and plays an active role in immune infiltration and immune response. The potential role of ITGB2 in tumor immunology in LGG provides new insight into the immunotherapy of LGG.

Aberrant DNA methylation plays an essential role in the occurrence and development of LGG (33). Therefore, we used the TCGALGG database to analyze the relationship between ITGB2 methylation and ITGB2mRNA expression and the clinical and prognostic role of ITGB2 methylation in LGG. It was found that there was a significant negative correlation between the expression of ITGB2mRNA and the Pearson coefficient of ITGB2 methylation in LGG tissues (r = -0.66). ITGB2 hypermethylation was positively correlated with good OS in patients with LGG, which is consistent with the excellent prognosis of LGG patients with low expression of ITGB2mRNA. Then, we further identified the CpG site of ITGB2 DNA promoter methylation. Nine ITGB2 DNA promoter methylation CpG sites were screened by univariate multivariate cox regression analysis, of which six CpG hypermethylation sites were positively correlated with a good prognosis of OS in LGG patients (p < 0.05). In order to better predict the survival of LGG patients, we used nine selected CpG loci to obtain the risk score (ITMRS) of ITGB2 methylation CpG loci. What is exciting is that ITMRS can better distinguish the OS (P of patients with LGG (< 0.0001). Moreover, ITMRS can better predict the three-year, five-year, and ten-year survival of patients with LGG (AUC values of 0.884, 0.767, and 0.704, respectively).

## Conclusion

To sum up, ITGB2 is negatively regulated by ITGB2 methylation. ITGB2 can be used as a potential marker of mesenchymal molecular subtypes of gliomas and as an independent predictive marker of OS in patients with malignant gliomas and provides new insights into the immunotherapy of LGG. The ITMRS obtained from the ITGB2 methylation CpG site can better predict the three-year, five-year, and ten-year survival of patients with LGG. These effects of ITGB2 are expected to bring the new gospel to patients’ survival, treatment, and prognosis with LGG.

## Data availability statement

Publicly available datasets were analyzed in this study. This data can be found here: (http://www.cgga.org.cn/) (http://gliovis.Bioinfo.cnio.es/) (https://www.cbioportal.org/).

## Author contributions

XH and CZ contributed to the study concepts, study design, and coordination responsibility for the research activity planning. HL and JW contributed to the data analysis and drafting of the manuscript, and implementation of the computer code and supporting algorithms. TL contributed to the critical advice on study design. ZZ, LL, and YZ contributed to the data analyses and interpretation. All authors contributed to the article and approved the submitted version.

## Supplementary material

Supplementary Figure 1 (A)The difference of ITGB2 methylation in IDH wt and mutation gliomas.(B)The difference of ITGB2 mRNA expression in MGMT-methylated and unmethylated gliomas.

Supplementary Figure 2 Kaplan-Meier curves of low and high ITGB2 DNA promoter CpG sites in LGG patients. cg01344119 **(A)**, cg19221500 **(B)**, cg02117924 **(C)**, cg18012089 **(D)**, cg17039369 **(E)**, cg13315706 **(F)**, cg20556503 **(G)**, cg21303803 **(H)**, cg07274406 **(I)**.

## References

[1] Louis DN. Molecular pathology of malignant gliomas. Annu Rev Pathol (2006) 1:97–117. doi: 10.1146/annurev.pathol.1.110304.100043 18039109

[2] Louis DN Ohgaki H Wiestler OD Cavenee WK Burger PC Jouvet A. The 2007 WHO classification of tumours of the central nervous system. Acta Neuropathol (2007) 114:97–109. doi: 10.1007/s00401-007-0243-4 17618441 PMC1929165

[3] Yang M Li Y Chilukuri K Brady OA Boulos MI Kappes JC. L1 stimulation of human glioma cell motility correlates with FAK activation. J Neurooncol (2011) 105:27–44. doi: 10.1007/s11060-011-0557-x 21373966 PMC3172337

[4] Jiang T Mao Y Ma W Mao Q You Y Yang X. CGCG clinical practice guidelines for the management of adult diffuse gliomas. Cancer Lett (2016) 375:263–73. doi: 10.1016/j.canlet.2016.01.024 26966000

[5] Stupp R Tonn JC Brada M Pentheroudakis G. High-grade malignant glioma: ESMO clinical practice guidelines for diagnosis, treatment and follow-up. Ann Oncol (2010) 21 Suppl 5:v190–3. doi: 10.1093/annonc/mdq187 20555079

[6] Goodenberger ML Jenkins RB. Genetics of adult glioma. Cancer Genet (2012) 205:613–21. doi: 10.1016/j.cancergen.2012.10.009 23238284

[7] Hayes J Yu Y Jalbert LE Mazor T Jones LE Wood MD. Genomic analysis of the origins and evolution of multicentric diffuse lower-grade gliomas. Neuro Oncol (2018) 20:632–41. doi: 10.1093/neuonc/nox205 PMC5892142 29077933

[8] Pals ST den Otter A Miedema F Kabel P Keizer GD Scheper RJ. Evidence that leukocyte function-associated antigen-1 is involved in recirculation and homing of human lymphocytes *via* high endothelial venules. J Immunol (1988) 140:1851–3. 3279123

[9] Rothlein R Dustin ML Marlin SD Springer TA. A human intercellular adhesion molecule (ICAM-1) distinct from LFA-1. J Immunol (1986) 137:1270–4. 3525675

[10] Marlin SD Springer TA. Purified intercellular adhesion molecule-1 (ICAM-1) is a ligand for lymphocyte function-associated antigen 1 (LFA-1). Cell (1987) 51:813–9. doi: 10.1016/0092-8674(87)90104-8 3315233

[11] Kijas JM Bauer TR Jr. Gäfvert S Marklund S Trowald-Wigh G Johannisson A. A missense mutation in the beta-2 integrin gene (ITGB2) causes canine leukocyte adhesion deficiency. Genomics (1999) 61:101–7. doi: 10.1006/geno.1999.5948 10512685

[12] Liu M Gou L Xia J Wan Q Jiang Y Sun S. LncRNA ITGB2-AS1 could promote the migration and invasion of breast cancer cells through up-regulating ITGB2. Int J Mol Sci (2018) 19 (7). doi: 10.3390/ijms19071866 PMC6073814 29941860

[13] Li J Zhang Z Feng X Shen Z Sun J Zhang X. Stanniocalcin-2 promotes cell EMT and glycolysis *via* activating ITGB2/FAK/SOX6 signaling pathway in nasopharyngeal carcinoma. Cell Biol Toxicol (2022) 38:2259–72. doi: 10.1007/s10565-021-09600-5 PMC8986754 33797657

[14] Kuppner MC Hamou MF de Tribolet N. Activation and adhesion molecule expression on lymphoid infiltrates in human glioblastomas. J Neuroimmunol (1990) 29:229–38. doi: 10.1016/0165-5728(90)90166-k 1698816

[15] Wu F Li GZ Liu HJ Zhao Z Chai RC Liu YQ. Molecular subtyping reveals immune alterations in IDH wild-type lower-grade diffuse glioma. J Pathol (2020) 251:272–83. doi: 10.1002/path.5468 32418210

[16] Wu F Wang ZL Wang KY Li GZ Chai RC Liu YQ. Classification of diffuse lower-grade glioma based on immunological profiling. Mol Oncol (2020) 14:2081–95. doi: 10.1002/1878-0261.12707 PMC7463381 32392361

[17] Zhang M Wang X Chen X Zhang Q Hong J. Novel immune-related gene signature for risk stratification and prognosis of survival in lower-grade glioma. Front Genet (2020) 11:363. doi: 10.3389/fgene.2020.00363 32351547 PMC7174786

[18] Deng X Lin D Zhang X Shen X Yang Z Yang L. Profiles of immune-related genes and immune cell infiltration in the tumor microenvironment of diffuse lower-grade gliomas. J Cell Physiol (2020) 235:7321–31. doi: 10.1002/jcp.29633 32162312

[19] Tang Z Li C Kang B Gao G Li C Zhang Z. GEPIA: a web server for cancer and normal gene expression profiling and interactive analyses. Nucleic Acids Res (2017) 45:W98–w102. doi: 10.1093/nar/gkx247 28407145 PMC5570223

[20] Gao J Aksoy BA Dogrusoz U Dresdner G Gross B Sumer SO. Integrative analysis of complex cancer genomics and clinical profiles using the cBioPortal. Sci Signal (2013) 6:l1. doi: 10.1126/scisignal.2004088 PMC4160307 23550210

[21] Li T Fan J Wang B Traugh N Chen Q Liu JS. TIMER: A web server for comprehensive analysis of tumor-infiltrating immune cells. Cancer Res (2017) 77:e108–10. doi: 10.1158/0008-5472.Can-17-0307 PMC6042652 29092952

[22] Carmona-Saez P Chagoyen M Tirado F Carazo JM Pascual-Montano A. GENECODIS: a web-based tool for finding significant concurrent annotations in gene lists. Genome Biol (2007) 8:R3. doi: 10.1186/gb-2007-8-1-r3 17204154 PMC1839127

[23] Verhaak RG Hoadley KA Purdom E Wang V Qi Y Wilkerson MD. Integrated genomic analysis identifies clinically relevant subtypes of glioblastoma characterized by abnormalities in PDGFRA, IDH1, EGFR, and NF1. Cancer Cell (2010) 17:98–110. doi: 10.1016/j.ccr.2009.12.020 20129251 PMC2818769

[24] Liu Z Han H He X Li S Wu C Yu C. Expression of the galectin-9-Tim-3 pathway in glioma tissues is associated with the clinical manifestations of glioma. Oncol Lett (2016) 11:1829–34. doi: 10.3892/ol.2016.4142 PMC4774531 26998085

[25] Stupp R Mason WP van den Bent MJ Weller M Fisher B Taphoorn MJB. Radiotherapy plus concomitant and adjuvant temozolomide for glioblastoma. N Engl J Med (2005) 352:987–96. doi: 10.1056/NEJMoa043330 15758009

[26] Lowenstein PR Baker GJ Castro MG. Cracking the glioma-NK inhibitory code: toward successful innate immunotherapy. Oncoimmunology (2014) 3:e965573. doi: 10.4161/21624011.2014.965573 25941594 PMC4292565

[27] Boussiotis VA Charest A. Immunotherapies for malignant glioma. Oncogene (2018) 37:1121–41. doi: 10.1038/s41388-017-0024-z PMC5828703 29242608

[28] Wu F Zhao Z Chai RC Liu YQ Li GZ Jiang HY. Prognostic power of a lipid metabolism gene panel for diffuse gliomas. J Cell Mol Med (2019) 23:7741–8. doi: 10.1111/jcmm.14647 PMC6815778 31475440

[29] Wang LM Li Z Piao YS Cai YN Zhang LY Ge HJ. Clinico-neuropathological features of isocitrate dehydrogenase 2 gene mutations in lower-grade gliomas. Chin Med J (Engl) (2019) 132:2920–6. doi: 10.1097/cm9.0000000000000565 PMC6964951 31833906

[30] Rajaraman P Brenner AV Butler MA Wang SS Pfeiffer RM Ruder AM. Common variation in genes related to innate immunity and risk of adult glioma. Cancer Epidemiol Biomarkers Prev (2009) 18:1651–8. doi: 10.1158/1055-9965.Epi-08-1041 PMC2771723 19423540

[31] Wang X Ning W Qiu Z Li S Zhang H Yu C. Tumor-associated macrophages based signaling pathway analysis and hub genes identification in glioma. Med (Baltimore) (2020) 99:e23840. doi: 10.1097/md.0000000000023840 PMC7748342 33371165

[32] Xu H Zhang A Han X Li Y Zhang Z Song L. ITGB2 as a prognostic indicator and a predictivemarker for immunotherapy in gliomas. Cancer Immunol Immunother (2022) 71(3):645–60. doi: 10.1007/s00262-021-03022-2 PMC10992927 34313821

[33] Mathur R Zhang Y Grimmer MR Hong C Zhang M Bollam S. MGMT promoter methylation level in newly diagnosed low-grade glioma is a predictor of hypermutation at recurrence. Neuro Oncol (2020) 22:1580–90. doi: 10.1093/neuonc/noaa059 PMC8444710 32166314

