# Supplementary figures and images for: Correlation between ITGB2 expression and clinical characterization of glioma and the prognostic significance of its methylation in low-grade glioma(LGG)
Source: Front Endocrinol (Lausanne). 2023 Jan 13;13:1106120. doi: 10.3389/fendo.2022.1106120 (PMC9880157; doi:10.3389/fendo.2022.1106120)

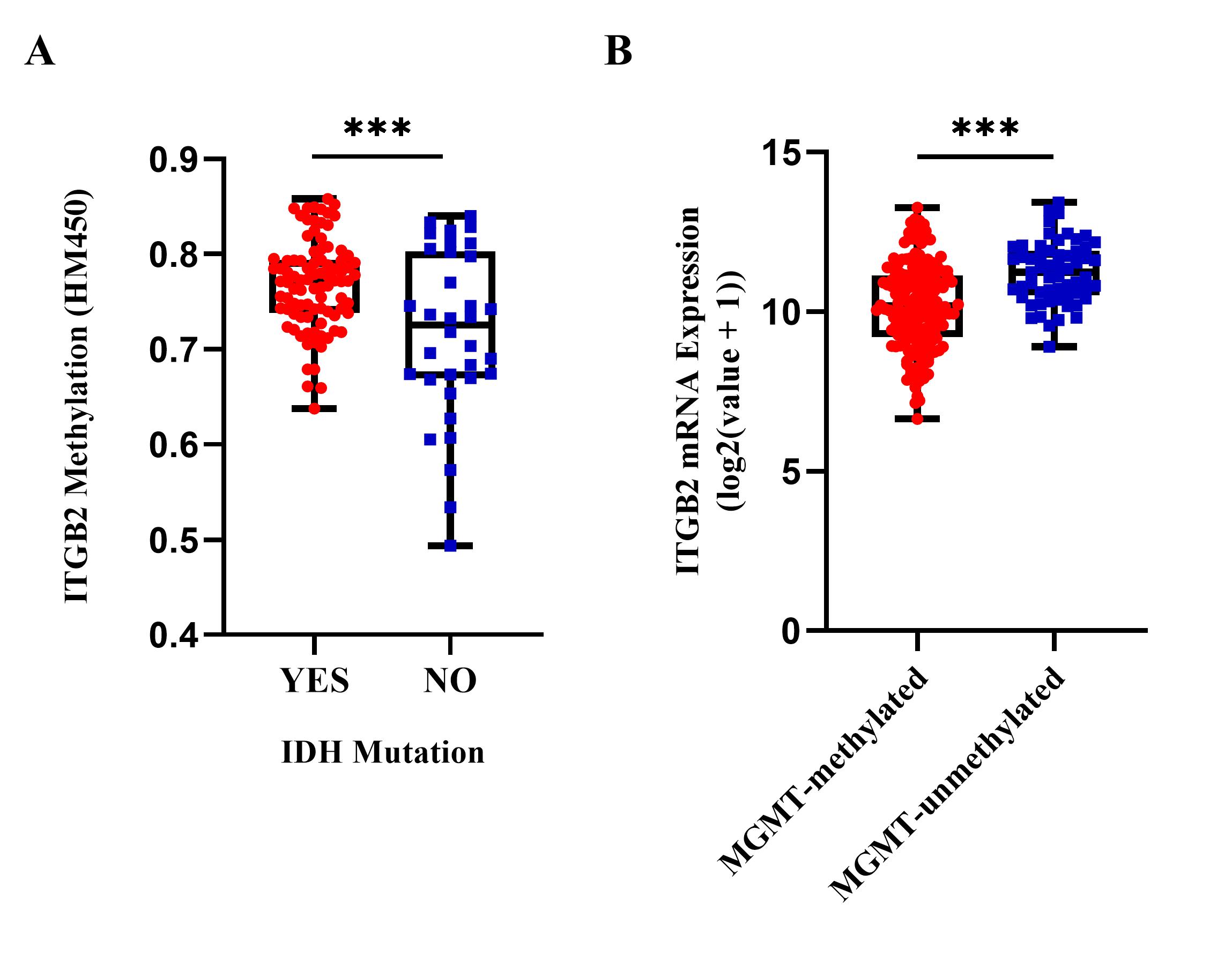

Supplement: Supplementary Figure 1 — (A)The difference of ITGB2 methylation in IDH wt and mutation gliomas.(B)The difference of ITGB2 mRNA expression in MGMT-methylated and unmethylated gliomas. [file Image_1.jpeg]

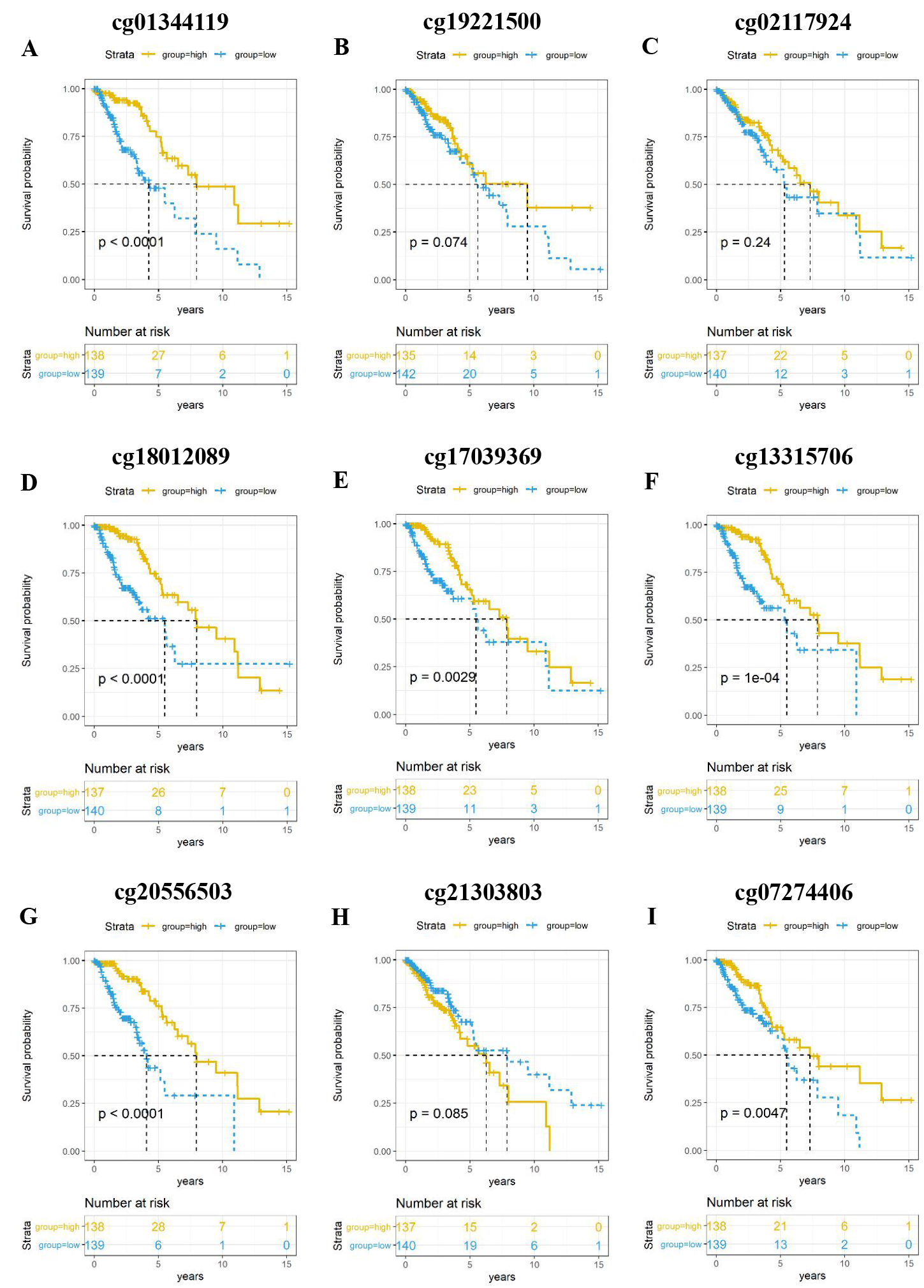

Supplement: Supplementary Figure 2 — Kaplan-Meier curves of low and high ITGB2 DNA promoter CpG sites in LGG patients. cg01344119 (A), cg19221500 (B), cg02117924 (C), cg18012089 (D), cg17039369 (E), cg13315706 (F), cg20556503 (G), cg21303803 (H), cg07274406 (I). [file Image_2.tif]
